# The associations between female fecundability and postpartum breastfeeding: A prospective cohort study

**DOI:** 10.1371/journal.pone.0331877

**Published:** 2025-09-05

**Authors:** Jiuming Li, Xiang Hong, Xiaoqi Zhu, Jingying Wu, Haitao Yang, Xuening Zhang, Bei Wang

**Affiliations:** 1 Key Laboratory of Environmental Medicine and Engineering of Ministry of Education, Department of Epidemiology and Health Statistics, School of Public Health, Southeast University, Nanjing, Jiangsu, China; 2 National Health Commission Contraceptives Adverse Reaction Surveillance Center, Nanjing, China; 3 Jiangsu Provincial Medical Key Laboratory of Fertility Protection and Health Technology Assessment, Nanjing, China; 4 Jiangsu Health Development Research Center, Nanjing, China; Wingate University, UNITED STATES OF AMERICA

## Abstract

**Objective:**

This study aimed to investigate the relationship between women’s fecundability and postpartum breastfeeding.

**Methods:**

We used a prospective cohort study design to recruit pregnant women who came to the hospital for antenatal checkups before 20 weeks’ gestation between April 2019 and March 2020 at the Maternal and Child Health Hospital of Gulou District, Nanjing, China. Women were categorized into prolonged time to pregnancy (TTP) group (>3 months) and shorten TTP (≤3 months) groups. A telephone follow-up was conducted 42 days postpartum to collect breastfeeding practice data. Logistic regression models were employed to analyze the association between TTP and exclusive breastfeeding.

**Results:**

A total of 535 pregnant women were initially included in the study cohort and 478(89.35%) completed the follow-up, among 79 (16.5%) in the prolonged TTP group, and the rest in the short TTP group (n = 399, 83.5%). According to the follow-up, 271 (56.7%) were in the exclusive breastfeeding group and the rest in the non-exclusive breastfeeding group (n = 207, 43.3%). A significant decrease in exclusive breastfeeding rate was observed in the prolonged TTP group compared to the short TTP group (OR=0.46, 95% CI: 0.27–0.74). After adjusting for potential confounders such as age, husband’s age, BMI, and regularity of menstruation, the negative association between TTP and exclusive breastfeeding remained (OR=0.50, 95% CI: 0.29–0.84). In stratified analyses, the results were generally consistent.

**Conclusion:**

The probability of postpartum breastfeeding is lower in women with lower fecundability. These findings suggest that women with lower fecundability may benefit from targeted breastfeeding support.

## Introduction

In recent years, countries around the world have responded to the World Health Organization’s (WHO) call to promote exclusive breastfeeding for approximately the first six months of life [1]. Breast milk is the best source of nutrition for an infant’s first six months, helping not only the infant’s digestion and growth but also the mother’s overall health. However, despite the many benefits of breastfeeding, China faces many challenges. On the one hand, changing social attitudes and the pressures of modern lifestyles have led more and more women to abandon breastfeeding in favor of artificial formula [2]. On the other hand, some mothers may face problems such as insufficient milk and mastitis, which affect the implementation of breastfeeding [3]. In addition, work pressure and a social environment that is not supportive of breastfeeding are among the difficulties faced in breastfeeding, with some women finding it difficult to obtain adequate support and facilities to continue breastfeeding at the workplace [4]. One report shows that [5] the exclusive breastfeeding rate for infants within 6 months of age in China is only 29.2%, which is significantly lower than the goal of more than 50% outlined in the Outline for the Development of Chinese Children (2021–2030).

Fecundability is the cornerstone of human reproduction, and it faces serious challenges as a result of social change and environmental impact. Safeguarding women’s ability to conceive is essential to maintaining a healthy and stable society. Waiting time to pregnancy (TTP) [6] is the interval between the initiation of uncontraceptive and regular sexual activity and the realization of pregnancy, and is a key indicator for fecundability assessment [7]. It is influenced by several factors, including age at menarche (AAM), menstrual regularity, menstrual period, menstrual cycle length, history of conception, history of adverse pregnancies, history of induced abortions, hypertension, diabetes mellitus, body mass index (BMI), and lifestyle choices such as tobacco use, alcohol consumption, and exposure to environmental toxins [8]. Several previous studies have suggested that the prolonged TTP group may be associated with an increased risk of future adverse pregnancy outcomes. A study analyzing 17,114 deliveries between 1989–2007 found that women with a TTP of 25–36 months had poorer neonatal health compared to women with a TTP of 0–6 months [9]. There is a significant relationship between TTP and pregnancy outcome and the prolonged TTP group may lead to adverse complications such as ectopic pregnancy, preterm labor, and miscarriage [10]. Emerging evidence suggests that women with prolonged TTP may face physiological challenges, such as hormonal imbalances (e.g., reduced prolactin or oxytocin levels), which could impair lactogenesis [10]. Additionally, behavioral factors, such as stress from prolonged conception efforts or differing attitudes toward breastfeeding, may influence postpartum practices [11]. Despite these potential links, no studies have explored the association between TTP and postpartum breastfeeding behavior.

We hypothesize that women with prolonged TTP may have a lower likelihood of exclusive breastfeeding due to physiological limitations in milk production or psychosocial factors, such as stress or reduced breastfeeding motivation. Understanding this relationship is critical for maternal and infant health, as breastfeeding is associated with reduced risks of chronic diseases in mothers and improved immune and cognitive development in infants [3]. This study aims to investigate the association between TTP and exclusive breastfeeding at 42 days postpartum using a prospective cohort design, providing evidence to support targeted breastfeeding interventions for women with lower fecundability.

## Materials and methods

### Characteristics of participants

This study used a prospective cohort design with the following inclusion criteria: (1) pregnant women who underwent prenatal checkups (gestational weeks ≤ 20 weeks) at the Maternal and Child Health Clinic of Gulou District, Nanjing, China, during the period of April 2019 to March 2020, with no selective recruitment.; (2) pregnant women who were conscious and able to communicate in written or verbal form; and (3) those who were willing to provide informed consent to participate in the study.

Exclusion criteria included pregnant women with medical contraindications to breastfeeding, such as HIV infection, active tuberculosis, use of medications incompatible with breastfeeding (e.g., chemotherapy agents).

**Fig 1:**
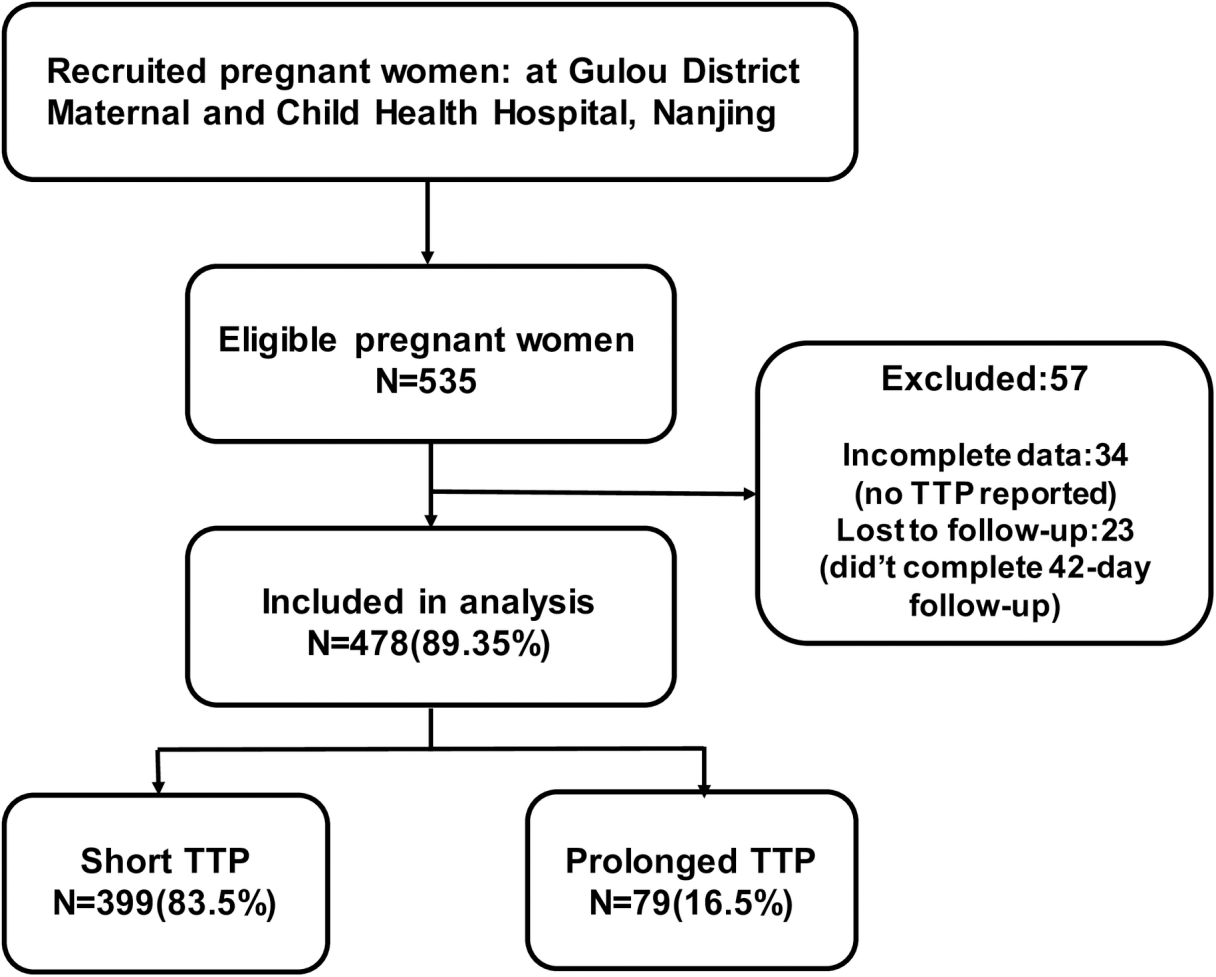
Participant Flowchart.

The cohort was not specifically designed for TTP but is a general mother-infant cohort aimed at investigating various maternal and infant health outcomes. For this particular analysis focusing on the association between TTP and postpartum breastfeeding, we applied additional criteria during the data analysis phase. Specifically, we excluded participants who did not report TTP and those who did not complete the 42-day postpartum follow-up and infants with conditions like galactosemia were excluded. (Fig 1)

The study was approved by the Ethics Committee of Zhongda Hospital, Southeast University (2018ZDSYLL116-P01). Informed consent was obtained from all subjects and/or their legal guardian(s). All data were fully anonymized.

### Baseline information collection

When pregnant women visited Gulou District Maternal and Child Health Hospital for antenatal checkups, uniformly trained investigators distributed questionnaires to pregnant women who met the enrollment criteria. Demographic information included the age and employment status of the pregnant woman and her husband, as well as the woman’s height, pre-pregnancy, and pregnancy weight; TTP was collected. Completion of the questionnaire was guided by the investigator.

### Definition of exposure

Following Tehrani et al., TTP was categorized as prolonged (>3 months) or short (≤3 months), a sensitive threshold for detecting fertility decline [10]. Although the diagnosis of infertility is 12 months of non pregnancy, using 3 months as the cut-off point for TTP extension can provide more sensitive reminders for early detection of fertility decline, thereby helping to seek medical intervention early. In addition, our research subjects were all pregnant women with a median TTP of less than 1 month. If we use 6 months as the cut-off point, it will result in too few participants in the TTP prolongation group, thereby affecting statistical efficacy. Women who conceived via assisted reproductive technologies (ART), such as in vitro fertilization (IVF), were included in the prolonged TTP group, as ART is typically pursued after prolonged unsuccessful natural conception attempts, reflecting lower fecundability. Women with unintended pregnancies were assigned to the short TTP group, as these pregnancies typically occur without extended conception efforts, indicating higher fecundability [11]. TTP was assessed based on self-reported uncontraceptive sexual activity to minimize misclassification from contraceptive failures.

### Outcome assessment

All enrolled subjects were followed up by telephone at 42 days postpartum, and a trained physician conducted a postpartum survey that included the mode of delivery, gestational week, neonatal status, and breastfeeding practices. Exclusive breastfeeding was defined as the provision of breast milk only, without the addition of any other liquids, solids, or water [12]. The rest of the definition is non-exclusive breastfeeding. This approach is standard in cohort studies to ensure complete data for the variables of interest. The 42-day postpartum follow-up was chosen as it aligns with standard postpartum checkups in China, a critical period for monitoring maternal and infant health and assessing breastfeeding establishment. This timepoint allows identification of early breastfeeding challenges, such as insufficient milk supply or return to work, which are common reasons for discontinuation.

### Statistical analysis

EpiData 3.1 software was used to create the database and to test the consistency of the double-entry program and the double-entry file. Statistical analysis was performed using R4.0.3. Non-normally distributed continuous variables were described by median and interquartile spacing, and the Wilcoxon rank sum test was used to compare differences between groups. Count data were expressed as frequencies and ratios, and comparisons between groups were made using the χ2 test. Multivariate logistic regression was used to analyze the association between TTP and the risk of exclusive breastfeeding at 42 days postpartum, and ORs and their 95% confidence intervals were estimated. A p-value of less than 0.05 indicated that the difference was statistically significant. A stratified analysis was conducted to assess the consistency of the association between TTP and exclusive breastfeeding across subgroups defined by female age, partner’s age, pre-pregnancy BMI, and menstrual regularity. These variables were selected due to their established influence on fertility and breastfeeding, as they may act as confounders or effect modifiers [13,14]. Stratification allowed exploration of potential variations in the association, ensuring robust findings and informing targeted interventions.

Our study adhered to the STROBE (Strengthening the Reporting of Observational Studies in Epidemiology) guidelines to ensure the rigor and comprehensiveness of our observational research.

## Results

### I. Participant characteristics

During the study period, a total of 535 pregnant women met the inclusion criteria, 23 cases were lost to follow-up, 34 cases had incomplete data, and finally, 478(89.35%) cases were included in the analysis. Univariate analysis showed significant differences between the short and the prolonged TTP groups in terms of age, husband’s age, education, pre-pregnancy body mass index (BMI), age at menarche, and menstrual regularity (p-value < 0.05) (Table 1). Specifically, maternal age below 35 years, husband’s age below 35 years, higher education, pre-pregnancy body mass index (BMI) of 18.5 to 24, earlier age at menarche, and regularity of menstrual cycle were associated with the short TTP group. However, no significant differences were observed in employment status, number of pregnancies, and menstrual cycle.

**Table 1:** Characteristics of participants.

Factors	Short TTP (percentage)(n = 399,83.5%)	Prolonged TTP (percentage)(n = 79,16.5%)	χ2 or W-value	P-value
Age (Mean±SD)	29 ± 4.26	31 ± 3.72	12446	<0.001
Husband’s age (Mean±SD)	30 ± 5.60	32 ± 4.33	12364	<0.001
BMI (Mean±SD)	21.04 ± 3.30	21.51 ± 3.28	12906	0.046
Age at menarche (Mean±SD)	13 ± 1.40	13 ± 1.31	18404	0.052
Menstrual cycle (days, Mean±SD)	30 ± 6.93	30 ± 6.05	15104	0.401
Age [years, n (%)]			1.43	0.231
<35	347 (86.97)	64 (81.48)		
≥35	52 (13.03)	15 (18.52)		
Husband’s age [years, n (%)]			5.4	0.02
<35	314 (78.70)	52 (65.82)		
≥35	85 (21.30)	27 (34.18)		
Employment Status. [n (%)]				
Employed	235 (58.90)	47 (59.49)	<0.001	1
Unemployed	164 (41.10)	32 (40.51)		
Educational level [n (%)]			5.45	0.065
Graduate students and above	53 (16.26)	10 (16.13)		
University	209 (64.11)	39 (62.90)		
High school and below	64 (19.63)	13 (20.97)		
Number of pregnancies [times, n (%)]			0.81	0.368
1	172 (43.11)	39 (49.37)		
≥2	227 (56.89)	40 (50.63)		
Pre-pregnancy body mass index [kg/m2, n (%)]			8.52	0.014
<18.5	53 (13.38)	9 (11.84)		
≥18.5~<24	287 (72.47)	46 (60.53)		
≥24	56 (14.14)	21 (27.63)		
Regularity of menstruation [n (%)]			5.72	0.017
Regularity (21–35 days)	359 (89.97)	63 (80.25)		
Irregularly	40 (10.03)	16 (19.75)		

### II. The associations between TTP and pregnancy outcome

In univariate analysis, no association was observed between TTP and the incidence of pregnancy complications such as gestational hypertension, gestational diabetes mellitus, and anemia (P > 0.05); and between TTP and pregnancy outcomes such as cesarean section and preterm delivery (P > 0.05). At the 42-day postpartum follow-up, the breastfeeding rate was higher in the short TTP group (59.90%) than in the prolonged TTP group (40.51%, p = 0.002) (Table 2).

**Table 2:** Comparative analysis of pregnancy complications and perinatal outcomes between short and prolonged TTP groups.

Factors	Short TTP (percentage)(n = 399)	Prolonged TTP (percentage)(n = 79)	χ2 or W-value	P-value
Spontaneous abortion [n (%)]		`	1.80E-28	1
Yes	2 (0.50)	0 (0)		
No	397 (99.50)	79 (100)		
Mode of delivery [n (%)]			1.05	0.305
Vaginal delivery	235 (58.90)	41 (51.90)		
Cesarean section	164 (41.10)	38 (48.10)		
Whether preterm labor [n (%)]			0.6	0.43
Premature labor	8 (2.01)	0 (0.00)		
full-term (gestation)	391 (97.99)	79 (100.00)		
Hypertension during pregnancy [n (%)]			0.09	0.76
Yes	16 (4.01)	2 (2.53)		
No	383 (95.99)	77 (97.47)		
Gestational diabetes mellitus [n (%)]			0.68	0.409
Yes	40 (10.02)	11 (13.93)		
No	359 (89.98)	68 (86.08)		
Anemia in pregnancy [n (%)]			0.01	0.919
Yes	131 (32.83)	27 (34.18)		
No	268 (67.16)	52 (65.82)		
Breastfeeding styles [n (%)]			9.33	0.002
Exclusive breastfeeding	239 (59.90)	32 (40.51)		
Non-exclusive breastfeeding	160 (40.10)	47 (59.49)		

### III. The associations between TTP and exclusive breastfeeding

In logistic regression analysis, the prolonged TTP group was associated with a 0.46 times lower likelihood of exclusive breastfeeding compared to the short TTP group (OR = 0.46, 95% CI: 0.27–0.74, p = 0.002). After adjusting for the influencing factors of age, husband’s age, BMI, regularity of menstruation, employment status, literacy, number of pregnancies, and age at menarche, the prolonged TTP group was still associated with exclusive breastfeeding (adjusted OR=0.48, 95% CI: 0.26–0.87, P = 0.017) (Table 3). This means that women in the prolonged TTP group had approximately half the odds of exclusively breastfeeding compared to those in the short TTP group, indicating a lower likelihood of exclusive breastfeeding.

**Table 3:** The associations between TTP and exclusive breastfeeding (Logistic regression).

	Crude OR	Model 1	Model 2
Short TTP	1	1	1
Prolonged TTP	0.46 (0.27~0.74)	0.50 (0.29-0.84)	0.48 (0.26-0.87)

Model1 was adjusted for female age, partner’s age, BMI and regularity of menstruation.

Model2 was adjusted for female age, partner’s age, BMI, regularity of menstruation, employment status, literacy and pregnancy age at menarche.

### IV. Stratified analysis

**Fig 2:**
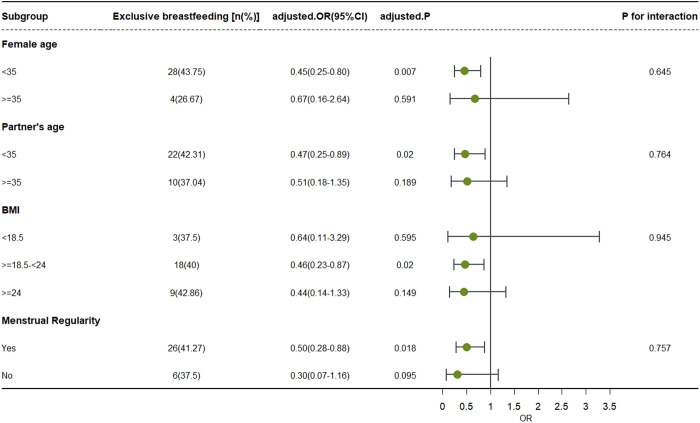
Stratified analyses.

Stratified analyses were performed to assess effect modification by variables such as women’s pre-pregnancy BMI, female age, partner’s age and menstrual regularity, selected due to their established associations with fecundability and breastfeeding in prior studies. There was a significant negative association between the prolonged TTP group and exclusive breastfeeding among pregnant women under 35 years of age (OR=0.45, 95% CI: 0.25–0.80). Similarly, there was a significant negative association between the prolonged TTP group and exclusive breastfeeding in husbands under 35 years of age (OR=0.47. 95% CI: 0.25–0.89, P = 0.02). Although this association was not statistically significant in the subgroup of older women and older husbands, there was no interaction between age and TTP (P = 0.645, for females, P = 0.764, for males), suggesting that the association was consistent across subgroups and that the result of no statistically significant association may be attributable to the low sample size within the subgroup. In addition, breastfeeding rates were lower among women with the prolonged TTP group among women with normal pre-pregnancy body mass index (OR=0.46, 95% CI: 0.23–0.87, P = 0.02). Breastfeeding rates were also lower among the prolonged TTP group of pregnant women with regular menstrual cycles (OR=0.50,95% CI: 0.28–0.88, P = 0.018). There was also no interaction between pre-pregnancy BMI, menstrual cycle regularity, and TTP (Fig 2).

## Discussion

The present study, utilizing a prospective cohort design, found that women with prolonged TTP (>3 months) had significantly lower odds of exclusive breastfeeding at 42 days postpartum compared to those with short TTP (≤3 months), with an adjusted OR of 0.48 (95% CI: 0.26–0.87) after controlling for confounders such as age, BMI, and menstrual regularity. This association persisted in stratified analyses, particularly among women under 35 years, those with husbands under 35 years, normal pre-pregnancy BMI, and regular menstrual cycles. Notably, no significant associations were observed between TTP and pregnancy complications (e.g., gestational hypertension, diabetes) or outcomes (e.g., cesarean section, preterm delivery).

Univariate analyses revealed that the short TTP group was characterized by younger maternal and paternal ages, higher education, normal BMI, earlier menarche, and regular menstruation, consistent with prior literature on fertility influencers [8,14]. For instance, Fang et al. (2020) reported similar associations between pre-pregnancy BMI and TTP in a Chinese cohort, highlighting how these factors may collectively enhance fecundability [14]. However, employment status, gravidity, and menstrual cycle length did not differ significantly between groups, suggesting these may play a lesser role in TTP variation in our population.

Contrary to some studies linking prolonged TTP to adverse pregnancy outcomes like preterm birth or miscarriage [9,10], our results showed no such associations. This discrepancy may stem from our cohort’s focus on early gestational recruitment and exclusion of high-risk cases, or from the relatively short median TTP in our sample (less than 1 month). These findings provide reassurance that prolonged TTP does not invariably predict complications in low-risk pregnancies.

The core finding of reduced exclusive breastfeeding in the prolonged TTP group aligns with emerging evidence on fertility and lactation. A meta-analysis by Tehrani et al. (2014) and more recent reviews indicate that prolonged TTP may reflect underlying endocrine issues affecting lactogenesis [10,15]. Stratified results further showed consistency across subgroups, with no significant interactions, reinforcing the robustness of this association [13,14].

Building on these results, the observed association may involve biological mechanisms, such as hormonal imbalances (e.g., lower prolactin/oxytocin) common in prolonged TTP or ART users, which impair milk production [10,15,16]. Psychosocial factors, including stress from conception delays or reduced breastfeeding confidence, could also contribute, as supported by qualitative studies on Chinese mothers facing work pressures [2,4,5]. These mechanisms are plausible given our data showing lower breastfeeding rates (40.51% vs. 59.90%) in the prolonged TTP group.

These findings have important implications for clinical practice, suggesting that women with prolonged TTP may benefit from targeted breastfeeding support, such as early lactation counseling or monitoring for milk supply issues.

This study has several limitations. First, the limited sample size may reduce generalizability, and excluding women without TTP data or follow-up may introduce selection bias. Second, recall bias in TTP reporting and subjective breastfeeding assessments may affect accuracy, warranting objective measures like preconception records or standardized tools (e.g., Breastfeeding Evaluation Scale) in future studies. Third, unassessed confounders, such as socioeconomic status, obstetric trauma, and postpartum coital activity, may influence the TTP-breastfeeding association. Finally, the study period (April 2019 onward) overlapped with the early COVID-19 pandemic, potentially affecting TTP or breastfeeding through stress, though adjustments for maternal health factors likely minimized confounding.

In conclusion, considering the above limitations, the findings of this paper need to be interpreted with caution. Future studies should aim to expand the sample size, reduce assessment bias, and use more objective and professional methods to conduct in-depth research on the relationship between breastfeeding and childbirth, to obtain a more comprehensive and reliable understanding of this important topic.

## Conclusion

This study concluded that women with lower fecundability may experience a decline in exclusive breastfeeding rates after delivery. In essence, the findings not only deepen the understanding of the intricate relationship between fecundability and breastfeeding preference but also provide a scientific basis for the development of targeted intervention strategies for different subgroups.

## Supporting information

S1 Table Sensitivity analysis. (XLSX)
